# Carbon, Nitrogen, and Phosphorus Allocation Strategy Among Organs in Submerged Macrophytes Is Altered by Eutrophication

**DOI:** 10.3389/fpls.2020.524450

**Published:** 2020-10-19

**Authors:** Qingyang Rao, Haojie Su, Xuwei Deng, Wulai Xia, Lantian Wang, Wenjian Cui, Linwei Ruan, Jun Chen, Ping Xie

**Affiliations:** ^1^Donghu Experimental Station of Lake Ecosystems, State Key Laboratory of Freshwater Ecology and Biotechnology, Institute of Hydrobiology, Chinese Academy of Sciences, Wuhan, China; ^2^University of Chinese Academy of Sciences, Beijing, China; ^3^Department of Ecology, College of Urban and Environmental Sciences, Peking University, Beijing, China; ^4^College of Resources and Environment, Anhui Agricultural University, Hefei, China; ^5^College of Life Sciences, Anhui Normal University, Wuhu, China

**Keywords:** nutrient allocation strategies, eutrophication, light and nutrient availability, submerged macrophyte, shallow lake

## Abstract

The allocation of limiting elements among plant organs is an important aspect of the adaptation of plants to their ambient environment. Although eutrophication can extremely alter light and nutrient availability, little is known about nutrient partitioning among organs of submerged macrophytes in response to eutrophication. Here, we analyzed the stoichiometric scaling of carbon (C), nitrogen (N), and phosphorus (P) concentrations among organs (leaf, stem, and root) of 327 individuals of seven common submerged macrophytes (three growth forms), sampled from 26 Yangtze plain lakes whose nutrient levels differed. Scaling exponents of stem nutrients to leaf (or root) nutrients varied among the growth forms. With increasing water total N (WTN) concentration, the scaling exponents of stem C to leaf (or root) C increased from <1 to >1, however, those of stem P to root P showed the opposite trend. These results indicated that, as plant nutrient content increased, plants growing in low WTN concentration accumulated leaf C (or stem P) at a faster rate, whereas those in high WTN concentration showed a faster increase in their stem C (or root P). Additionally, the scaling exponents of stem N to leaf (or root) N and stem P to leaf P were consistently large than 1, but decreased with a greater WTN concentration. This suggested that plants invested more N and P into stem than leaf tissues, with a higher investment of N in stem than root tissues, but eutrophication would decrease the allocation of N and P to stem. Such shifts in plant nutrient allocation strategies from low to high WTN concentration may be attributed to changed light and nutrient availability. In summary, eutrophication would alter nutrient allocation strategies of submerged macrophytes, which may influence their community structures by enhancing the competitive ability of some species in the process of eutrophication.

## Introduction

Anthropogenic activity and industrial development have extremely altered the availability of nitrogen (N) and phosphorus (P) in terrestrial and freshwater ecosystems across the globe (Dodds et al., 2009; Rockstrom et al., 2009; Marklein and Houlton, 2012; Penuelas et al., 2013). Changing nutrient availability is predicted to influence structure and function of ecosystems, because N and P, being essential nutrient elements for plant growth, are generally considered to be limiting resources in nature (Koerselman and Meuleman, 1996; Elser et al., 2010). Eutrophication arising from N and P enrichments is supposed to reduce light availability by promoting the growth of phytoplankton and periphyton, thereby resulting in a shading effect upon submerged macrophytes (Roberts et al., 2003; Sayer et al., 2010; Olsen et al., 2015). Furthermore, the allocation of limiting resources, as an important adaptive strategy for how plants respond to different and changeable environments, reflects the influences of evolutionary history, environmental stresses and trade-offs of functional traits (Xie et al., 2005; Kerkhoff et al., 2006; Dulger et al., 2017; Scartazza et al., 2017; Li et al., 2019). To maximize their multiple functions, namely growth, reproduction, and nutrient storage of plants, plants need to rebalance the allocation of elements across organs under various environmental stresses (Schreeg et al., 2014; Yang et al., 2014). However, considerable attention has focused on the nutrient allocation strategies of terrestrial plants (Yang et al., 2014; Yan et al., 2016; Zhao et al., 2019), relatively little is known about aquatic plants.

Submerged macrophytes, being important primary producers in shallow lakes, play a vital role in maintaining a clear water state and ecosystem functioning (Jeppesen et al., 1998; Scheffer et al., 2001). According to their phenotype, submerged vegetation can be classified into four types of growth forms: canopy former, erect, rosette, and bottom dweller (Chambers and Kalff, 1987). Each form has adapted to different habitats in terms of light availability and nutrient status, indicating species-specific resource utilization and allocation strategies. For instance, canopy formers (as well as erect species) tend to elongate the shoot to alleviate conditions of low light stress, while rosette species usually produce longer and wider leaves to enhance their photosynthetic ability (Strand and Weisner, 2001; Schneider et al., 2015; Chen et al., 2016). Such adjustments in morphological characteristics entail nutrient reallocation from the whole plant, such that nutrient concentrations across plant organs are tightly coordinated. Scaling relationship analysis provides a useful tool for exploring the association of nutrient contents across organs that may reveal the nutrient allocation strategies for aquatic plants. The scaling approach, Y = β X^α^, is often used to examine the relationships in plant traits, where X and Y are pairs of traits, β is the regression intercept, and α is the regression slope (i.e., scaling exponent) (Enquist, 2002). Scaling relationships are widely applied to explore plant nutrient allocation, mainly in two ways: nutrient partitioning within a specific organ for different nutrients, and nutrient partitioning among organs for the same nutrient. Overall, previous studies have mainly focused on the nutrient concentrations of different tissues (Scartazza et al., 2017; Velthuis et al., 2017; Chou et al., 2019) and biomass allocation among organs (Xie et al., 2005). By contrast, current knowledge of nutrient allocation patterns among organs is extremely limited for submerged macrophytes.

The leaves, stems, and roots of aquatic vascular plants function differentially to compose and maintain an organic whole. Leaves are the main organs of photosynthesis, whereas stems not only let plants endure the hydrodynamic force arising from waves but also transport minerals and carbohydrates within them (Steward, 1964; Sorrell, 2004; Schutten et al., 2005), while roots can store non-structural carbohydrates and absorb mineral salts, nutrients, and water from the local environment (Bristow and Whitcombe, 1971; Dulger et al., 2017). Reproductive organs maintain higher nutrient contents to stimulate seedling establishment and propagation (Fenner, 1986; Kerkhoff et al., 2006). Carbon (C) allocated to leaves forms the main part of photosynthetic products, whereas N and P are the key constituents of photosynthetic apparatuses (Marschner and Marschner, 2012). The C allocated to stems is used to form structural compounds (mainly lignin and structural polysaccharides) and non-structural carbohydrates, while N and P play pivotal roles in photosynthate loading and transportation in the phloem (Marschner and Marschner, 2012; Grasset et al., 2015). In addition, submerged macrophytes can absorb nutrients via both their shoots and roots, and excessive uptake of elements may be observed in these tissues (Carignan and Kalff, 1980; Sterner and Elser, 2002).

Nutrient allocation strategies among organs may reflect the interplay between plants and their ambient environments, because plants can coordinate different organs to satisfy the nutritional requirements of various functions with respect to the allocation of limited nutrients and adaptation to a changing environment (Liu et al., 2010). According to previous studies, plant species as well as the availability of light resources and nutrient can significantly influence the nutrient concentrations of a single organ (Xing et al., 2013; Grasset et al., 2015; Su et al., 2016; Dulger et al., 2017; Li et al., 2017, 2019; Velthuis et al., 2017). Accordingly, we hypothesize that nutrient allocation among organs differs not only among growth forms but also changes with key environmental factors. Our hypotheses are as follows: (1) scaling relationships among organs differ across different macrophyte growth forms. (2) as plant nutrient content increases, plants living in low water total N (WTN) concentrations show a higher allocation to leaf C content, whereas those in high WTN concentrations show a higher allocation to stem C content. (3) nutrient and light availabilities together drive the pattern of plant nutrient allocation changing along WTN gradients. Here, we test these hypotheses by researching how the growth forms, water and sediment nutrients, and ambient light condition jointly affect the scaling relationships of stem nutrients to leaf (or root) nutrients of seven common submerged macrophyte species in 26 Yangtze floodplain shallow lakes.

## Materials and Methods

### Study Sites and Filed Sampling

**FIGURE 1:**
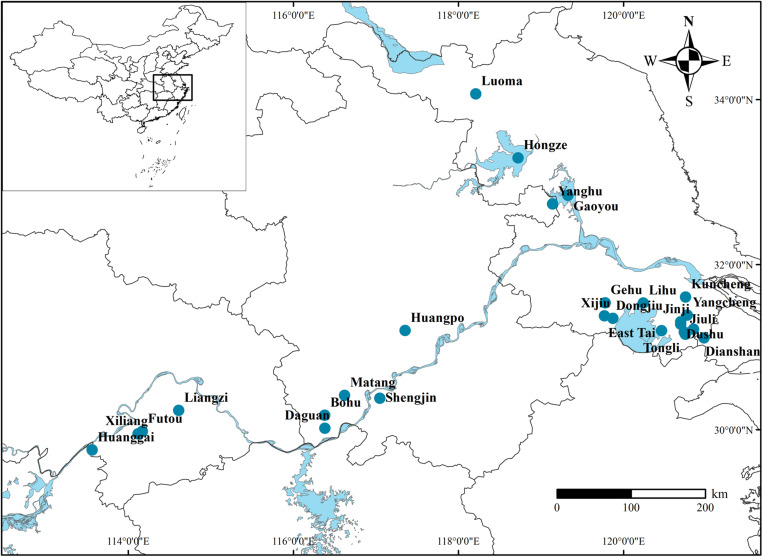
Locations of 26 sampled lakes in the Yangtze floodplain.

This study was conducted in 26 lakes along the middle and lower reaches of the Yangtze river during the growing season (June–September) in 2018 (Figure 1). The Yangtze floodplain, one of the three largest plains in China, contains multitudinous shallow lakes with various nutrient levels (Deng et al., 2020). The lake area and average water depth ranged from 2.1 to 2537.2 km^2^ and 1.03 to 3.25 m, respectively (Supplementary Table 1 for details). In recent years, nitrogen’s role in the decline of aquatic plants has garnered more attention and concern (Jeppesen et al., 2007; Moss et al., 2013). Therefore, we divided all sampled individuals into five subgroups based on the lakes’ WTN to examine the effects of eutrophication on the relationships of nutrient content among plant organs (WTN ≤ 0.5, 0.5 < WTN ≤ 1, 1 < WTN ≤ 1.5, 1.5 < WTN ≤ 2, and WTN > 2 mg L^–1^ for TN-1, TN-2, TN-3, TN-4, and TN-5, respectively). According to Chinese environmental quality standards for surface water in 2002, WTN concentrations of 0.5, 1, 1.5, and 2 mg L^–1^ correspond to II, III, IV, and V type of water, respectively (General Administration of Quality Supervision, Inspection, and Quarantine of the People’s Republic of China [AQSIQ], 2002).

In this study, we focused on seven species of common submerged macrophytes widely distributed in lakes of the Yangtze plain, each of which could be classified into one of three types of growth forms: canopy former, erect and rosette (Chambers and Kalff, 1987) (canopy former: *Potamogeton maackianus*, *Potamogeton wrightii*, *Ceratophyllum demersum*, and *Myriophyllum spicatum*; erect: *Potamogeton crispus* and *Hydrilla verticillate*; rosette: *Vallisneria natans*). Submerged macrophytes were collected using a rotatable reaping hook covering 0.2 m^2^ in belt transect of macrophytic region, with three times at each site. The plants were carefully washed, sorted by species, and then divided into leaf, stem and root parts. Corresponding sediment samples and water samples were respectively collected from the top layers of sediment and at 0.5 m below the water surface. These samples were stored in portable fridges and taken to the laboratory for further measurements. The leaves and stems were collected from aboveground parts of plants, but stems of *Vallisneria natans* were stolons buried into sediment. The roots of individuals were considered those tissues situated below the sediment surface, but the roots of *Ceratophyllum demersum* were absent. In total, we sampled 327 individuals of the seven species from the 26 Yangtze lakes.

### Laboratory Measurement

The samples of aquatic plants and sediments were first dried at 80°C to a constant weight and then ground into fine powder before their elemental analysis. For total C and total N (TN) concentration, the samples were determined using an element analyzer (Flash EA 1112 series, CE Instruments, Italy). After the sediment and plant samples were digested respectively using H_2_SO_4_–HClO_4_ and H_2_SO_4_–H_2_O_2_, their total P (TP) concentrations were measured by the ammonium molybdate ascorbic acid method (Sparks et al., 1996). TN and TP concentrations in water samples were analyzed as nitrate and ortho-phosphate after performing a K_2_S_2_O_8_ digestion; for more details, standard methods can be referred to (Huang et al., 1999). The Secchi depth (SD) was measured with a Secchi disk. Chl-a in water column was extracted using the ethanol method after filtration through a Whatman GF/F filter and then quantified by a spectrophotometer (Jespersen and Christoffersen, 1987).

### Data Analysis

Stoichiometric scaling relationships of C, N, and P among the plant organs were examined using this equation: log_10_ Y = α × (log_10_X) + β, where X and Y represent leaf (or root) C (or N, or P) and stem C (or N, or P), and the exponents α and β are the regression slope (i.e., scaling exponent) and the regression intercept, respectively. Reduced major axis (RMA) was used to determine the parameters α and β after log_10_-transforming the variables. When α > 1, it meant that Y changed faster than linearly with X, whereas α < 1 indicated that X increased faster than linearly with Y. We conducted these analyses in three ways. First, we divided the plant individual data into three growth forms (canopy former/erect/rosette) and five WTN levels (TN-1/TN-2/TN-3/TN-4/TN-5), and then compared their respective scaling exponents. Second, we performed scaling analyses for each WTN level, and related these scaling exponents to local environmental factors (sediment TN, sediment TP, WTN, water TP, and SD) of each WTN level, by using linear regression. Third, we compared scaling exponents of canopy former species from low to high WTN concentrations. Note that in this research we did not perform detailed analyses on the scaling exponents of erect or rosette species at the five WTN levels because this data was lacking. Likelihood ratio tests were used to determine significant differences in RMA scaling exponents among different growth forms and WTN levels. One-way ANOVA (post hoc Turkey HSD) was applied to investigate the differences in each environmental factors among the five WTN level, implemented in SPASS 22.0. Linear regressions were performed to explore the pattern of scaling exponents’ change with environmental factors. The RMA analysis and linear regressions were conducted using the R packages “smatr” and “stats,” respectively (R Core Team, 2018).

## Results

### Scaling Relationships of C, N and P Among Leaves, Stems, and Roots Across Plant Growth Forms

**FIGURE 2:**
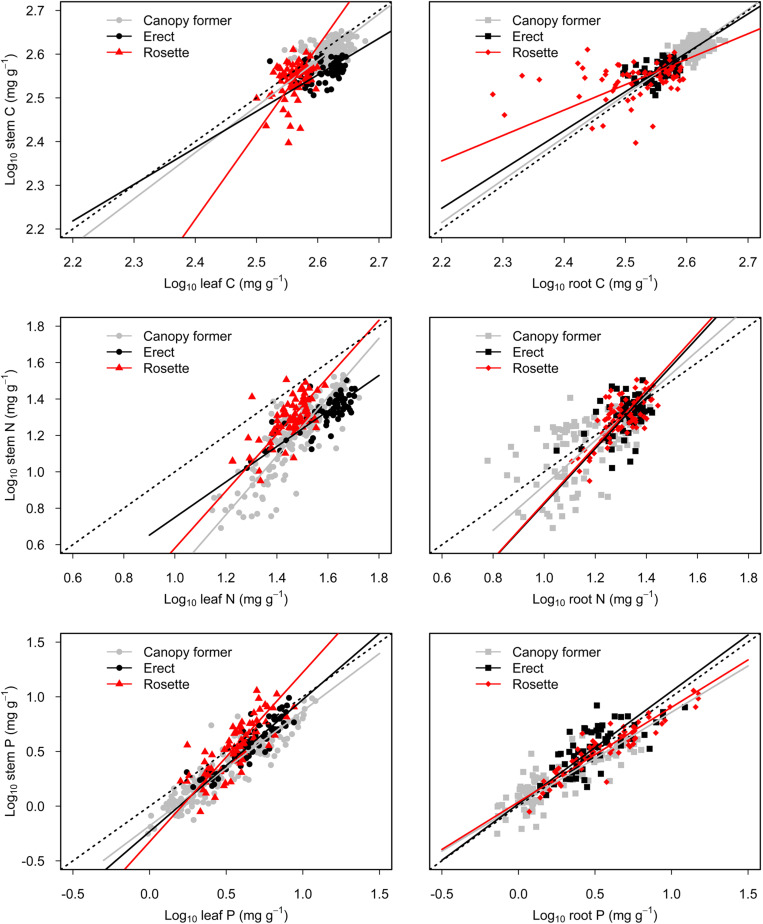
Scaling relationships of stem nutrients to leaf (or root) nutrients for submerged macrophytes grouped by three growth forms (canopy former/erect/rosette). Reduced major axis (RMA) regression was applied to determine the significant line (*p* < 0.05).

There were significant scaling relationships between stem nutrients and leaf (or root) nutrients, and their scaling exponents varied among growth forms except for that of stem P to root P [i.e., α_P_ (S-R)] (Table 1 and Figure 2). Scaling exponents of stem C (or N, or P) to leaf C (or N, or P) [i.e., α_C_ (S-L), or α_N_ (S-L), or α_P_ (S-L)] were significantly higher in rosette plants [1.98, 1.56, and 1.55 for α_C_ (S-L), α_N_ (S-L) and α_P_ (S-L), respectively]. Pooling the data from three growth forms, the α_C_ (S-L), α_N_ (S-L), and α_P_ (S-L) were all larger than 1 [1.21, 1.43, 1.19 for α_C_ (S-L), α_N_ (S-L), and α_P_ (S-L), respectively] (Table 1 and Figure 2). For all three growth forms, their scaling exponents of stem C to root C [i.e., α_C_ (S-R)] were smaller than 1 (0.98, 0.89, and 0.58 for canopy former, erect, and rosette, respectively), whereas those of stem N to root N [i.e., α_N_ (S-R)] were larger than 1 (1.23, 1.52, and 1.54 for canopy former, erect, and rosette, respectively) (Table 1 and Figure 2).

**TABLE 1:** Summary of reduced major axis (RMA) regression results for nutrient allocation among organs [e.g., log_10_stem C (or N, or P) = α × log_10_ leaf (or root) C (or N, or P) + β].

	*n*	α_RMA_ (95% CI)	β_RMA_(95% CI)	*r*^2^	*p*
**Scaling exponents of stem (C, or N, or P) to leaf (C, or N, or P)**					
C					
All	325	1.21 (1.11; 1.32)	−0.57 (−0.84; −0.30)	0.39	<0.001
Growth form					
Canopy former	184	1.06b (0.93; 1.20)	−0.16 (−0.52; 0.20)	0.20	<0.001
Erect	68	0.84b (0.66; 1.05)	0.38 (−0.13; 0.89)	0.11	0.006
Rosette	73	1.98a (1.58; 2.47)	−2.52 (−3.66; −1.39)	0.10	0.007
N					
All	324	1.43 (1.34; 1.54)	−0.89 (−1.04; −0.74)	0.59	<0.001
Growth form					
Canopy former	183	1.62a (1.49; 1.75)	−1.17 (−1.36; −0.99)	0.71	<0.001
Erect	67	0.97b (0.84; 1.13)	−0.22 (−0.46; 0.01)	0.63	<0.001
Rosette	74	1.56a (1.31; 1.87)	−0.98 (−1.39; −0.58)	0.42	<0.001
P					
All	317	1.19 (1.13; 1.25)	−0.21 (−0.25; −0.18)	0.78	<0.001
Growth form					
Canopy former	180	1.05c (0.99; 1.11)	−0.18 (−0.21; −0.15)	0.84	<0.001
Erect	67	1.21b (1.09; 1.34)	−0.23 (−0.32; −0.15)	0.82	<0.001
Rosette	70	1.55a (1.33; 1.80)	−0.33 (−0.46; −0.19)	0.61	<0.001
**Scaling exponents of stem (C, or N, or P) to root (C, or N, or P)**					
C					
All	247	0.75 (0.69; 0.82)	0.65 (0.47; 0.82)	0.49	<0.001
Growth form					
Canopy former	113	0.98a (0.82; 1.15)	0.07 (−0.36; 0.50)	0.19	<0.001
Erect	62	0.89a (0.71; 1.12)	0.29 (−0.24; 0.82)	0.19	<0.001
Rosette	72	0.58b (0.46; 0.73)	1.08 (0.74; 1.41)	0.09	0.013
N					
All	248	1.28 (1.17; 1.40)	−0.37 (−0.51; −0.22)	0.49	<0.001
Growth form					
Canopy former	112	1.23b (1.06; 1.43)	−0.31 (−0.52; −0.09)	0.38	<0.001
Erect	62	1.52a (1.21; 1.91)	−0.70 (−1.16; −0.23)	0.19	<0.001
Rosette	74	1.54a (1.31; 1.82)	−0.71 (−1.04; −0.38)	0.50	<0.001
P					
All	231	0.94 (0.88; 0.99)	0.01 (−0.02; 0.04)	0.78	<0.001
Growth form					
Canopy former	106	0.85a (0.76; 0.94)	0.01 (−0.02; 0.04)	0.73	<0.001
Erect	60	1.03a (0.85; 1.24)	0.02 (−0.09; 0.13)	0.49	<0.001
Rosette	65	0.87a (0.79; 0.95)	0.04 (−0.01; 0.09)	0.86	<0.001

*Different letters indicate that scaling exponents are significantly different (*p* < 0.05) based on likelihood tests.*

### Scaling Relationships of C, N, and P Among Leaves, Stems, and Roots Across WTN Concentrations of Lakes

**FIGURE 3:**
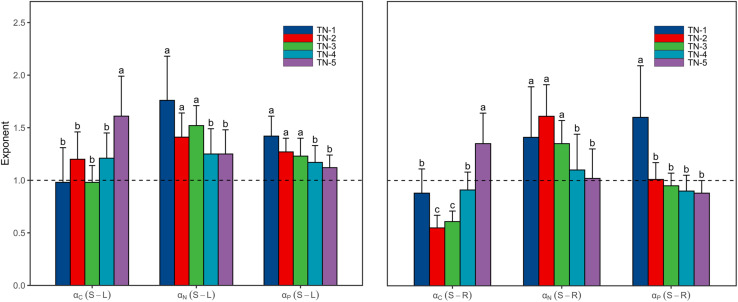
The scaling exponents, α_C_ (S-L/R), α_N_ (S-L/R) and α_P_ (S-L/R), change along the WTN concentrations (WTN ≤ 0.5, 0.5 < WTN ≤ 1, 1 < WTN ≤ 1.5, 1.5 < WTN ≤ 2, and WTN > 2 mg L^–1^ for TN-1, TN-2, TN-3, TN-4, and TN-5, respectively). The bar charts and error bars display the scaling exponents and 95% confidence interval (CI). Different letters indicate that scaling exponents are significantly different (*p* < 0.05) based on likelihood tests.

As the WTN concentrations increased, scaling exponents for C [α_C_ (S-L/R)] increased from <1 in low WTN concentrations to >1 in high WTN concentrations, whereas those for N and P [α_N_ (S-L/R) and α_P_ (S-L/R)] decreased with an increasing WTN concentration (Figure 3 and Supplementary Table 2). In addition, both the α_N_ (S-L/R) and α_P_ (S-L) were larger than 1 at each WTN level, but α_P_ (S-R) significantly decreased from >1 in low WTN concentrations (WTN < 0.5 mg L^–1^) to <1 in high WTN concentrations (WTN > 2 mg L^–1^) (Figure 3 and Supplementary Table 2).

### Relationships Between Scaling Exponents and Environmental Factors

**FIGURE 4:**
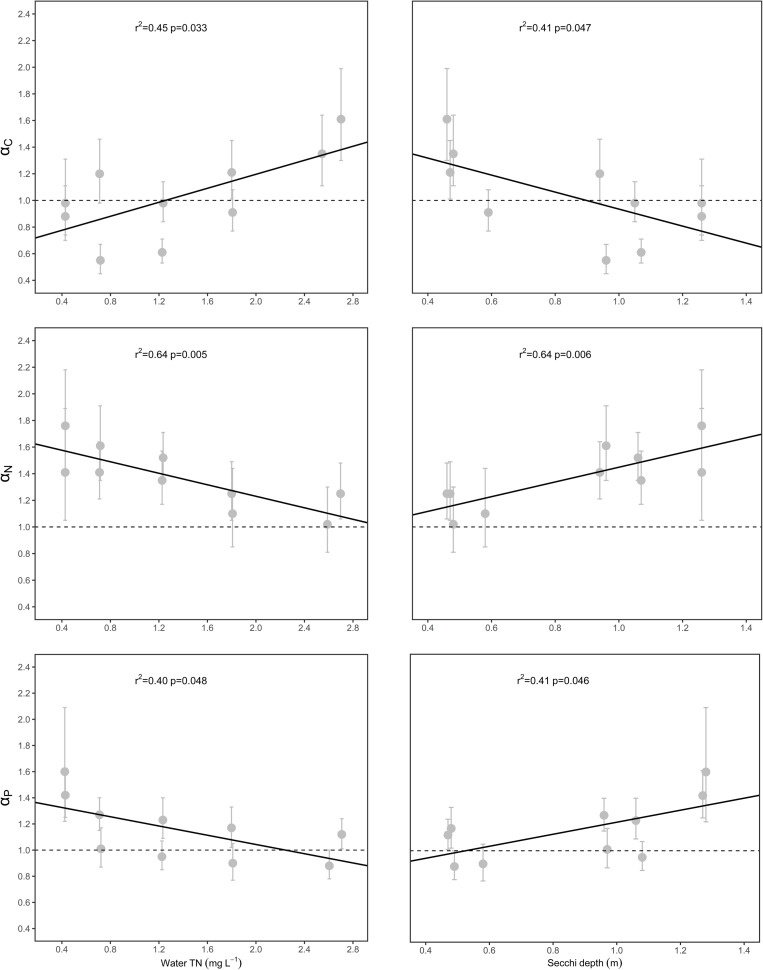
Relationships between scaling exponents (α_C_, α_N_, and α_P_) and environmental factors. Points and error bars display the scaling exponents and 95% confidence interval (CI), and linear regression (*p* < 0.05) is used to fit to the exponents.

The α_C_ [i.e., α_C_ (S-L/R)], α_N_ [i.e., α_N_ (S-L/R)], and α_P_ [i.e., α_P_ (S-L/R)] values were significantly related to WTN concentrations and SD (*p* < 0.05), but only weakly correlated with sediment TN, sediment TP, and water TP concentrations (Figure 4 and Supplementary Figure 1). Specifically, α_C_ increased with increasing WTN concentrations, but it decreased with increasing SD, yet the opposite trend characterized both α_N_ and α_P_ (Figure 4 and Supplementary Table 3).

## Discussion

### Different Nutrient Allocation Strategies Among Organs in Three Plant Growth Forms

The higher α_C_ (S-L), α_N_ (S-L), and α_P_ (S-L) in rosette than either canopy former or erect (Table 1) indicated that, as plant nutrients increased, the rosette species had a faster increase in stem nutrient than did the other two growth forms. In this study, the rosette species is *vallisneria natans*, and its stem is stolon that is an asexual reproductive organ. *Vallisneria natans* is an important aquatic clonal plant in China, whose clonal growth seems to be an essential way by which to expand its natural population (Xiao et al., 2007). For reproductive growth, nutrients are reallocated from vegetative organs to reproductive tissues; hence the latter contain higher nutrients contents to promote seedlings’ establishment, propagation and population development (Fenner, 1986; Kerkhoff et al., 2006; Lambers et al., 2008). Compared with canopy former and erect species, rosette species might require greater investment in stolon to meet the higher demand imparted by clonal growth, which is related to the increased partitioning of nutrients to stems. Furthermore, the α_C_ (S-L), α_N_ (S-L), and α_P_ (S-L) were larger than 1 when the data are pooled together (Table 1), which demonstrated that, as plant nutrients (C, N, and P) increased, submerged macrophytes generally underwent a faster increase in stem nutrients. Due to strong water currents and waves in shallow lakes, submerged macrophytes may experience low light conditions in terms of strong sediment resuspension induced by waves in shallow lakes (Zheng et al., 2015; Bertrin et al., 2017). Additionally, nutrient enrichment in water column promotes the growth of phytoplankton and periphyton, thereby resulting in low light conditions in eutrophic lakes (Roberts et al., 2003; Sayer et al., 2010; Olsen et al., 2015). Our sampled lakes are shallow lakes and most have high concentration of nutrients and low light availability in water column (Supplementary Table 1 for details). Submerged macrophytes tend to elongate their stem to alleviate low light stress (Chen et al., 2016). According then, it is reasonable to think they might allocate more nutrients into structural compounds of stems during the shoot elongation phase. Further, stem plays an indispensable role by connecting the aboveground and belowground part of plants. Therefore, submerged macrophytes may require more N and P in their phloem loading and export apparatus to satisfy their internal demand for nutrient transportation (Marschner and Marschner, 2012; Grasset et al., 2015), which would be associated with a higher investment of nutrients to stem tissues.

For all three growth forms, both α_C_ (S-R) and α_P_ (S-R) (except for erect) were smaller than 1, but α_N_ (S-R) was larger than 1 (Table 1). These results suggested submerged macrophytes allocated more C and P to roots and invested more N in their stems as plant nutrient concentrations increased. Root is a storage organ that can accumulate non-structural carbohydrates for future later use under nutrient limiting conditions and for plant recruitment in spring (Dulger et al., 2017), which is associated with a higher C demand by root. As is well known, both shoots and roots of aquatic plants can take up nutrients, and the main source of nutrients for submerged macrophytes depends on the relative concentrations of nutrients in the sediment and water column (Carignan and Kalff, 1980). Relatively abundant N resources in water column and higher P contents in sediment typically occur in the habitat of these three growth forms, as shown in Supplementary Table 6. It follows that, roots are able to absorb more P due to relatively higher P concentrations in sediment (Carignan and Kalff, 1980; Sterner and Elser, 2002), which in our study was correlated with higher P investment in root. Concerning N, a large amount of it in the water column may attenuate excessive uptake of N by roots. Besides, stems play an important role in photosynthate loading, and so they might need more N to sustain its phloem transport.

### Light and Nutrient Availability Affect Nutrient Allocation Strategies Among Organs in Plants Under Different WTN Levels

**FIGURE 5:**
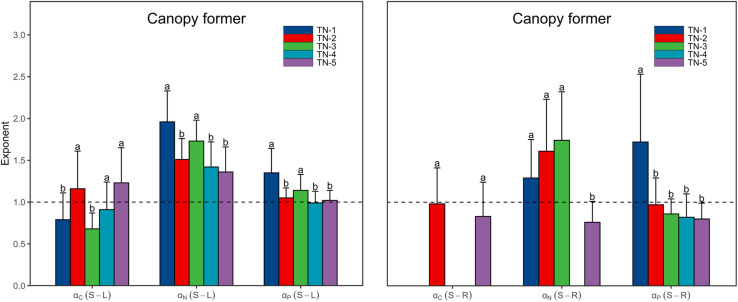
The scaling exponents, α_C_ (S-L/R), α_N_ (S-L/R), and α_P_ (S-L/R), for canopy former change along the WTN concentrations (WTN ≤ 0.5, 0.5 < WTN ≤ 1, 1 < WTN ≤ 1.5, 1.5 < WTN ≤ 2, and WTN > 2 mg L^–1^ for TN-1, TN-2, TN-3, TN-4, and TN-5, respectively). The bar charts and error bars display the scaling exponents and 95% confidence interval (CI). Different letters indicate that scaling exponents are significantly different (*p* < 0.05) based on likelihood tests. The missing bar charts indicate the relationships between stem and root nutrient contents are statistically non-significant.

Both α_C_ (S-L) and α_C_ (S-R) increased from <1 to >1 with increasing WTN concentrations, whereas the α_P_ (S-R) showed the opposite trend (Figure 3 and Supplementary Table 2). This result indicated that, as plant nutrient contents increased, submerged macrophytes growing in low WTN concentrations tended toward a higher increase in leaf and root C contents and stem P content, whereas submerged macrophytes in high WTN concentrations tended toward a higher increase in stem C and root P contents. Additionally, we found that, α_N_ (S-L), α_N_ (S-R), and α_P_ (S-L) were always larger than 1, but decreased nonetheless with increasing WTN concentrations (Figure 3 and Supplementary Table 2). This suggested that, with increasing plant nutrient contents, a higher investment of N and P in stem than leaf parts and a higher investment of N in stem than root occurred for plants from low to high WTN concentrations. However, eutrophication would decrease the allocation of N and P to stem tissues. The scaling exponents of canopy former species [except for α_C_ (S-R)] presented similar patterns along the WTN gradient (Figure 5 and Supplementary Table 4). Such patterns of scaling exponents changing with WTN concentrations may be partly attributable to differences in growth forms because of the dominance of canopy former at each WTN level (Supplementary Figure 2). In addition, similar growth forms between TN-1 and TN-5 levels led to distinct scaling exponents (Figure 3 and Supplementary Figure 2). Thus, minor changes in species composition might modestly contribute to the aforementioned patterns of nutrient allocation strategies among organs.

The relationships between scaling exponents and five environmental factors were also explored in this study. We found that WTN and SD could be the main drivers for the aforementioned patterns (Figure 4 and Supplementary Figure 1), since both factors had significant relationships with α_C_ (S-L/R), α_N_ (S-L/R), and α_P_ (S-L/R). Generally, water clarity and light availability increase with an increasing SD. Previous studies have demonstrated that both nutrient and light availability could influence the C, N, and P concentrations of plant tissues (Cronin and Lodge, 2003; Xing et al., 2013; Su et al., 2016; Dulger et al., 2017; Velthuis et al., 2017). As WTN concentrations increased and SD decreased, the α_C_ (S-L/R) increased from <1 to >1 (Figure 4); this suggested that, as plant nutrient content increased, plants in low water turbidity and WTN concentrations underwent a higher increase in their leaf and root C contents, whereas a higher increase in stem C content characterized plants growing in high water turbidity and WTN concentrations. This divergent allocation pattern could have several explanations. First, robust photosynthetic performance under low WTN concentrations is positively associated with higher water transparency and light availability (Supplementary Table 3) (Chen et al., 2016), so such plants might be better able to produce more carbohydrates in leaves and allocate more C to their roots for storage. A combination of high light and low nutrient concentration has shown to lead to an increase of non-structural carbohydrates in plant tissues (Huber et al., 2012). By contrast, low light availability and low photosynthetic activity in low SD but high WTN concentrations likely reduced the production of carbohydrates in leaves and hence the allocation of C to roots. Second, since canopy former and erect species tend to elongate their shoots to capture the light under low light conditions (Strand and Weisner, 2001; Fu et al., 2012; Schneider et al., 2015), this requires more C investment in their stems. For rosette species, their clonal growth is inhibited under conditions of low light stress, so their stem (i.e., stolon, an asexual reproductive organ) may keep more carbohydrates in reserve for re-growth and propagation under suitable environmental conditions.

Both α_N_ (S-L/R) and α_P_ (S-L/R) decreased with increasing WTN concentrations and a decreasing SD (Figure 4), yet the α_N_ (S-L/R) and α_P_ (S-L) were larger than 1 (Figure 3 and Supplementary Table 2). High photosynthetic activity in low water turbidity and WTN concentrations can promote the production of more carbohydrates, which correspondingly demands more N and P in the photosynthate loading and export apparatus of phloem. Carbohydrate accumulation in leaves can result in a negative feedback adjustment of photosynthesis under high irradiance, which may cause declines in chlorophyll and RubisCO contents and saved nitrogen in terrestrial plants (Long et al., 2004; Leakey et al., 2009). By contrast, low photosynthetic ability of plants in high water turbidity and WTN concentrations inhibits their formation of C, which may attenuate the photosynthate transport and nutrient requirements of phloem in submerged macrophytes. However, more N and P might be allocated to leaves so as to offset the diminished activity of photosynthesis induced by low light. In addition, the shoots and roots of submerged macrophytes can uptake and store too much non-necessary N and P under nutrient enrichment conditions (Carignan and Kalff, 1980; Sterner and Elser, 2002). These phenomena could explain our results showing that eutrophication lessened the allocation of N and P to the stem. Given the crucial function of stems in connecting the leaves and roots and transporting nutrients and photosynthates, plants showed a higher investment of N and P in stem than leaf parts and a higher investment of N in stem than in root in the process of eutrophication. Sediment TP significantly increased with the increase of WTN concentrations, but water TP did not (Supplementary Table 3). Therefore, roots absorbed and stored more P vis-a-vis the higher P contents in sediment, which could explain the faster increase in root P that was found here under higher WTN concentrations of lakes.

### Further Studies of Nutrient Partitioning Among Organs

Sediment nutrients and water TP might be potential drivers for plant nutrient allocation strategies changing with ambient environment, considering their prominent participation in shaping ecological stoichiometric characteristics of plants (Li et al., 2018; Su et al., 2018). However, we found that the relationships of scaling exponents to sediment nutrients or water TP were all statistically non-significant (Supplementary Figure 1). Lake sediment is widely considered to harbor much spatial heterogeneity (Hilton and Gibbs, 1984; Mackay et al., 2012), and sediment nutrients at each WTN level may therefore not directly reflect the bioavailability of elements for an entire lake. Furthermore, because these environmental factors are closely correlated (Supplementary Table 5), distinguishing their independent effects on nutrient allocation is a difficult task. Thus, more controlled experiments are needed to explore their singular effects on the partitioning of nutrients among organs in aquatic plants.

Plant organs are interconnected with each other, functioning to coordinate and affect the whole-plant performance of species in different environments. Both nutrient concentration and organ biomass can influence the allocation of nutrients among organs. Therefore, information on organ biomass should also be included in further investigations of nutrient partitioning and corresponding analyses. Ideally, future research would evaluate morphological traits (such as leaf area, plant height, and specific stem [root] length), photosynthesis and respiration rates, physiological processes (non-structural carbohydrates and other C–N metabolic indicators) as well as reproductive characteristics (seed mass and seed number), to construct the trait correlation network at the whole-plant level to illustrate comprehensively the adaptation of plants to various environmental factors. Our 26 sampled lakes are located in the middle and lower reaches of Yangtze floodplain, where the average annual temperature and precipitation is 14–18°C and 1,000–1,400 mm, respectively (Chen et al., 2018). Small thermal and moisture gradients in this region limit the exploration of latitudinal patterns of nutrient allocation strategies among organs on a large spatial scale. Yet we do know that temperature is the key driver for stoichiometric patterns of aquatic macrophytes in eastern China (Xia et al., 2014). Additionally, for the submerged macrophytes in our study, their scaling exponents are more “variable” in terms of the changed nutrient allocation relationships along the examined environmental gradients. Therefore, large-scale studies are imperative for revealing further details about the interactions between aquatic plants and their ambient environments. It bears mentioning that our lakes are all shallow lakes, whose water depth ranges from 1.03 to 3.25 m. Strong water movement can affect shoot elongation and plant development directly, and thereby indirectly influence the growth of submerged macrophytes via sediment resuspension (Kankanamge et al., 2011; Bertrin et al., 2017). Similarly, fish disturbance inhibits the growth of these plants by increasing nutrient loading in the water column via sediment resuspension (Cu et al., 2018; Chen et al., 2020). These phenomena may result in changes to both light and nutrient availability given the strong sediment resuspension that occurs in shallow lakes. Therefore, further research on the effects of wave and fish upon nutrient portioning should be pursued using fine-scale experiments.

## Conclusion

Our results indicate that plant growth forms and environmental factors affect the nutrient partitioning among organs, and that light and nutrient availability drive a gradual shift in plant nutrient allocation strategies when going from low to high WTN concentrations across a large nutrient gradient in Yangtze floodplain lakes. The variation in nutrient allocation among organs across different WTN levels can be interpreted by “optimal partitioning theory” (Gedroc et al., 1996), which proposes that plants would allocate their limiting resources to optimize growth and obtain a “functional equilibrium” for adaptation to different environmental conditions. Eutrophication alters the nutrient allocation strategy of submerged macrophytes, which may affect the community structures of these aquatic plants by enhancing the competitive ability of some species, perhaps leading to their local dominance. Also, these patterns for nutrient allocation changing with WTN gradients are dependent upon WTN and SD, which could broaden our knowledge about the “light-nutrient hypothesis”: it argues that a balance between light and nutrient controls aquatic ecosystem processes and structures (Sterner et al., 1997). Our results reveal the partitioning of nutrients among macrophytes’ organs is more “variable” than “constant” because of the changes to scaling relationships between plants and environmental factors. Overall, from the perspective of stoichiometric scaling of nutrients among organs, nutrient allocation strategies are evidently altered by eutrophication, a finding that could broaden our understanding of how plants adapt to the environment as well as plant-environment interactions during the process of eutrophication.

## Data Availability Statement

The datasets generated for this study are available on request to the corresponding author.

## Author Contributions

JC and PX designed the study. WX, LW, WC, and LR assisted with the field sampling and laboratory measurement. QR, HS, and XD analyzed the data. QR wrote the manuscript. JC, PX, HS, and XD revised the manuscript. All authors approved the final manuscript.

## Conflict of Interest

The authors declare that the research was conducted in the absence of any commercial or financial relationships that could be construed as a potential conflict of interest.
