# Supplementary material for: Carbon, Nitrogen, and Phosphorus Allocation Strategy Among Organs in Submerged Macrophytes Is Altered by Eutrophication
Source: Front Plant Sci. 2020 Oct 19;11:524450. doi: 10.3389/fpls.2020.524450 (PMC7604295; doi:10.3389/fpls.2020.524450)
Supplement: Supplementary file 1 [file Table_1.DOC]

**Supplementary Information**

**Supplementary Figures S1-S2**

**Supplementary Tables S1-S6**

**Supplementary Figures**

**Supplementary Figure 1** Relationships between scaling exponents (α_C_, α_N_ and α_P_) and environmental factors. Points and error bars display the scaling exponents and 95% confidence interval (CI), and linear regression (p < 0.05) is used to fit to the exponents.


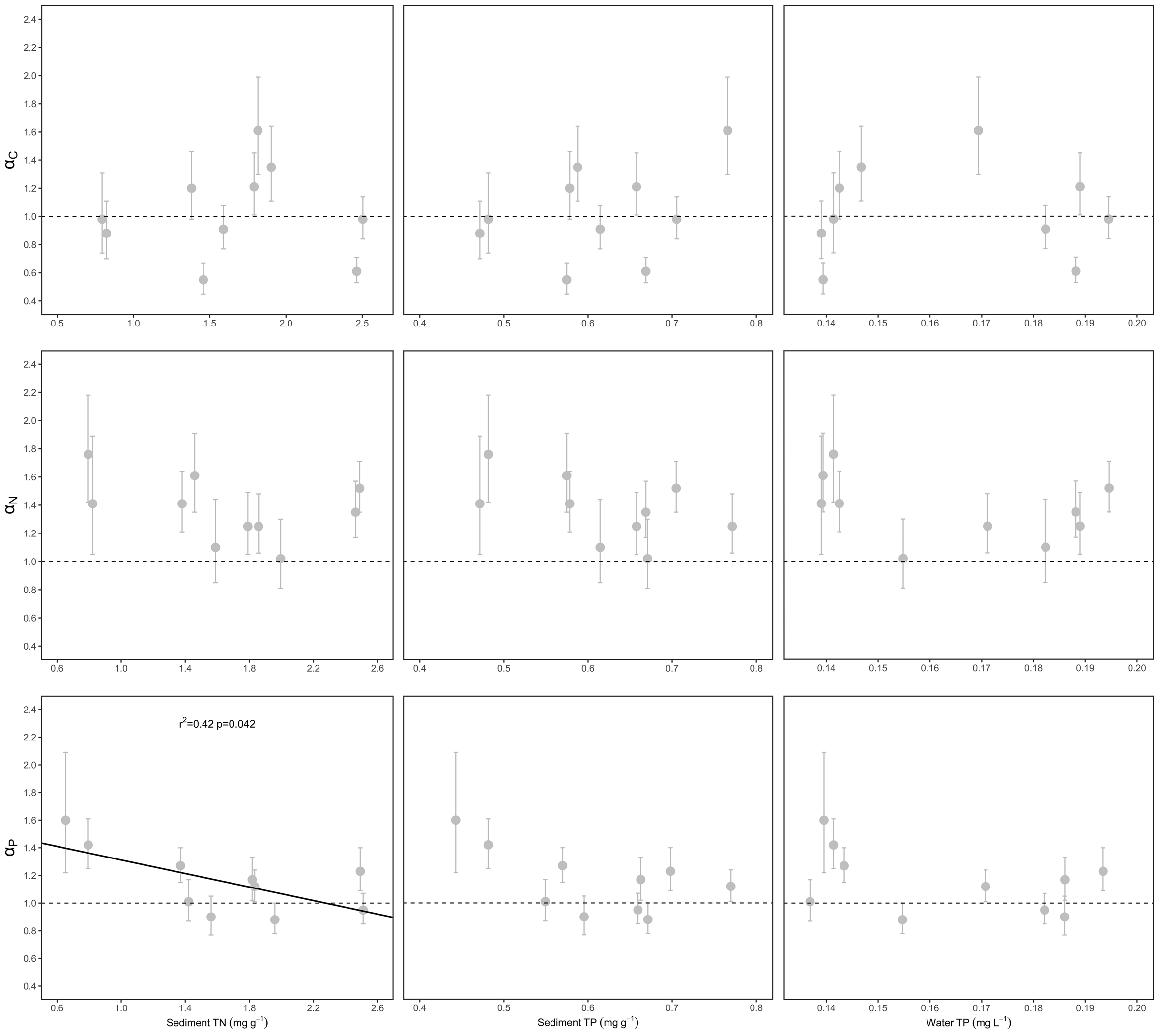


**Supplementary Figure 2** Growth forms at each WTN level (WTN≤0.5, 0.5<WTN≤1, 1<WTN≤1.5, 1.5<WTN≤2 and WTN>2 mg L^-1^ for TN-1, TN-2, TN-3, TN-4 and TN-5, respectively).


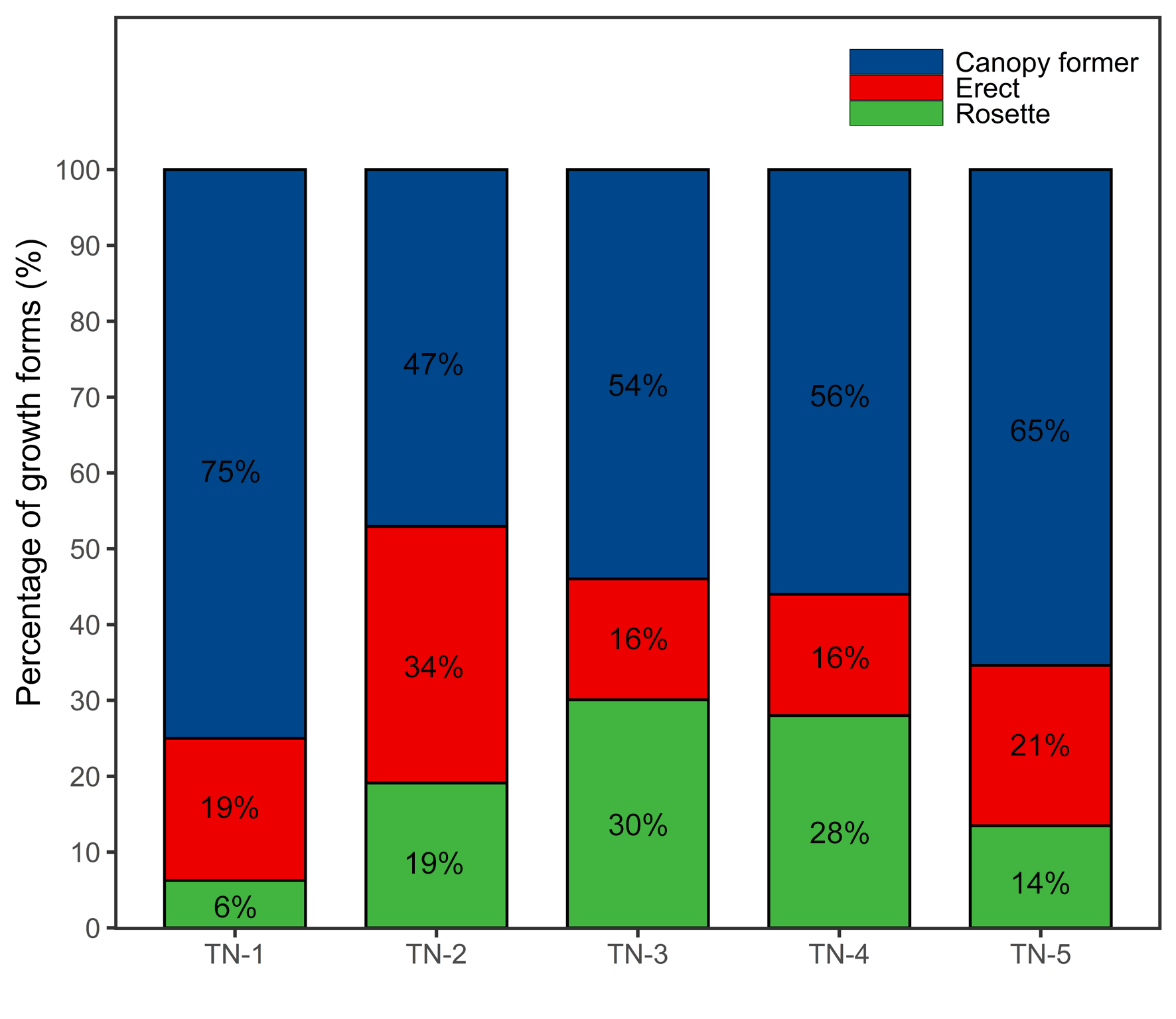


**Supplementary Tables**

**Supplementary Table 1** Geographic and trophic parameters of the 26 lakes in the Yangtze floodplain. WD, water depth; SD, Secchi depth; Chl-a, chlorophyll a.

| Lake | Latitude | Longitude | Area  (km^2^) | Sediment TN  (mg g^-1^) | Sediment TP  (mg g^-1^) | Water TN  (mg L^-1^) | Water TP  (mg L^-1^) | WD  (m) | SD  (m) | Chl-a  (μg L^-1^) | |
| --- | --- | --- | --- | --- | --- | --- | --- | --- | --- | --- | --- |
| Bohu Lake | 30.15 | 116.45 | 127.0 | 1.88 | 0.70 | 1.23 | 0.14 | 2.63 | 0.73 | 14.65 |  |
| Chenghu Lake | 31.21 | 120.82 | 45.0 | 1.26 | 0.92 | 2.02 | 0.21 | 2.78 | 0.50 | 25.51 |  |
| Daguan Lake | 30.03 | 116.33 | 146.0 | 2.47 | 0.74 | 1.54 | 0.16 | 2.23 | 0.88 | 26.16 |  |
| Dianshan Lake | 31.12 | 120.96 | 62.0 | 1.21 | 0.82 | 1.53 | 0.27 | 1.82 | 0.71 | 48.23 |  |
| Dongjiu Lake | 31.35 | 119.87 | 7.8 | 0.87 | 1.21 | 2.91 | 0.23 | 1.48 | 0.17 | 31.25 |  |
| Dushu Lake | 31.27 | 120.70 | 9.2 | 1.46 | 1.25 | 1.29 | 0.23 | 2.80 | 0.68 | 62.22 |  |
| Futou Lake | 30.02 | 114.20 | 114.2 | 2.76 | 1.29 | 2.42 | 0.22 | 1.53 | 0.57 | 41.85 |  |
| Gehu Lake | 31.61 | 119.82 | 146.5 | 1.56 | 1.10 | 2.02 | 0.30 | 1.56 | 0.16 | 44.83 |  |
| Gaoyou Lake | 32.85 | 119.35 | 639.2 | 1.07 | 0.68 | 1.44 | 0.29 | 1.13 | 0.17 | 15.41 |  |
| Huangpo Lake | 31.18 | 117.39 | 37.9 | 0.68 | 0.29 | 1.25 | 0.12 | 1.40 | 1.25 | 14.42 |  |
| Huanggai Lake | 29.71 | 113.54 | 86.0 | 2.39 | 0.60 | 2.13 | 0.23 | 1.77 | 0.43 | 58.59 |  |
| Hongze Lake | 33.29 | 118.69 | 1576.9 | 2.07 | 0.38 | 1.86 | 0.15 | 1.27 | 0.33 | 12.09 |  |
| Jinji Lake | 31.31 | 120.70 | 6.8 | 1.53 | 0.90 | 1.01 | 0.24 | 2.67 | 0.43 | 39.95 |  |
| Jiuli Lake | 31.19 | 120.73 | 2.1 | 1.69 | 1.07 | 1.44 | 0.19 | 1.60 | 0.50 | 12.13 |  |
| Kuncheng Lake | 31.59 | 120.75 | 18.0 | 0.72 | 0.95 | 0.95 | 0.18 | 2.85 | 0.46 | 8.97 |  |
| Lihu Lake | 31.51 | 120.26 | 9.1 | 1.22 | 0.73 | 0.80 | 0.19 | 2.99 | 0.40 | 39.46 |  |
| Luoma Lake | 34.04 | 118.21 | 290.9 | 2.25 | 0.48 | 2.45 | 0.12 | 3.25 | 0.73 | 23.27 |  |
| Liangzi Lake | 30.23 | 114.59 | 351.8 | 2.64 | 0.49 | 1.88 | 0.15 | 2.09 | 0.56 | 56.39 |  |
| Matang Lake | 30.42 | 116.62 | 7.0 | 1.63 | 0.48 | 1.34 | 0.18 | 1.03 | 0.24 | 16.34 |  |
| Shengjin Lake | 30.39 | 117.09 | 132.8 | 1.97 | 0.81 | 1.17 | 0.21 | 2.5 | 0.5 | 22.79 |  |
| Taihu Lake | 31.20 | 120.46 | 2537.2 | 0.67 | 0.38 | 0.57 | 0.15 | 1.55 | 1.35 | 3.21 |  |
| Tongli Lake | 31.16 | 120.73 | 2.9 | 1.34 | 1.11 | 1.64 | 0.25 | 1.55 | 0.25 | 25.86 |  |
| Xijiu Lake | 31.39 | 119.74 | 11.3 | 0.90 | 1.06 | 3.26 | 0.22 | 1.57 | 0.15 | 14.01 |  |
| Xiliang Lake | 29.98 | 114.11 | 98.1 | 5.19 | 0.59 | 1.16 | 0.14 | 2.03 | 2.03 | 3.50 |  |
| Yangcheng Lake | 31.43 | 120.76 | 119.0 | 1.39 | 0.66 | 0.90 | 0.19 | 1.42 | 0.71 | 26.71 |  |
| Yanghu Lake | 32.73 | 119.14 | 6.3 | 0.95 | 0.53 | 1.68 | 0.18 | 1.1 | 0.2 | 10.46 |  |

**Supplementary Tables**

**Supplementary Table 2** Summary of reduced major axis (RMA) regression results for nutrient allocation among organs (e.g., log_10_stem C (or N, or P) = α*log_10_ leaf (or root) C (or N, or P) + β) of submerged macrophytes grouped by five WTN levels (WTN≤0.5, 0.5<WTN≤1, 1<WTN≤1.5, 1.5<WTN≤2 and WTN>2 mg L^-1^ for TN-1, TN-2, TN-3, TN-4 and TN-5, respectively). Different letters indicate that scaling exponents are significantly different (p< 0.05) based on likelihood tests.

|  | **n** | **αRMA (95%CI)** | **βRMA（95%CI）** | **r^2^** | **p** |
| --- | --- | --- | --- | --- | --- |
| Scaling exponents of stem (C, or N, or P) to leaf (C, or N, or P) | | | | | |
| C | | | | | |
| All | 325 | 1.21(1.11; 1.32) | -0.57(-0.84; -0.30) | 0.39 | <0.001 |
| TN level | | | | | |
| TN-1 | 32 | 0.98b(0.74; 1.31) | 0.04(-0.70; 0.79) | 0.39 | <0.001 |
| TN-2 | 72 | 1.20b(0.98; 1.46) | -0.54(-1.17; 0.10) | 0.28 | <0.001 |
| TN-3 | 115 | 0.98b(0.84; 1.14) | 0.02(-0.36; 0.41) | 0.34 | <0.001 |
| TN-4 | 52 | 1.21b(1.01; 1.45) | -0.56(-1.14; 0.01) | 0.59 | <0.001 |
| TN-5 | 54 | 1.61a(1.30; 1.99) | -1.61(-2.51; -0.71) | 0.40 | <0.001 |
| N | | | | | |
| All | 324 | 1.43(1.34; 1.54) | -0.89(-1.04; -0.74) | 0.59 | <0.001 |
| TN level | | | | | |
| TN-1 | 32 | 1.76a(1.42; 2.18) | -1.33(-1.88; -0.78) | 0.67 | <0.001 |
| TN-2 | 72 | 1.41a(1.21; 1.64) | -0.86(-1.19; -0.54) | 0.59 | <0.001 |
| TN-3 | 116 | 1.52a(1.35; 1.71) | -1.04(-1.31; -0.76) | 0.57 | <0.001 |
| TN-4 | 52 | 1.25b(1.05; 1.49) | -0.62(-0.95; -0.28) | 0.61 | <0.001 |
| TN-5 | 52 | 1.25b(1.06; 1.48) | -0.58(-0.90; -0.25) | 0.64 | <0.001 |
| P | | | | | |
| All | 317 | 1.19(1.13; 1.25) | -0.21(-0.25; -0.18) | 0.78 | <0.001 |
| TN level | | | | | |
| TN-1 | 32 | 1.42a(1.25; 1.61) | -0.25(-0.32; -0.18) | 0.88 | <0.001 |
| TN-2 | 68 | 1.27a(1.15; 1.40) | -0.26(-0.33; -0.18) | 0.84 | <0.001 |
| TN-3 | 113 | 1.23a(1.09; 1.40) | -0.24(-0.34; -0.15) | 0.56 | <0.001 |
| TN-4 | 51 | 1.17b(1.02; 1.33) | -0.23(-0.32; -0.14) | 0.79 | <0.001 |
| TN-5 | 53 | 1.12b(1.01; 1.24) | -0.17(-0.24; -0.09) | 0.87 | <0.001 |
| Scaling exponents of stem (C, or N, or P) to root (C, or N, or P) | | | | | |
| C | | | | | |
| All | 247 | 0.75(0.69; 0.82) | 0.65(0.47; 0.82) | 0.49 | <0.001 |
| TN level | | | | | |
| TN-1 | 25 | 0.88b(0.70; 1.11) | 0.32(-0.20; 0.84) | 0.72 | <0.001 |
| TN-2 | 63 | 0.55c(0.45; 0.67) | 1.19(0.90; 1.47) | 0.39 | <0.001 |
| TN-3 | 85 | 0.61c(0.53; 0.71) | 1.00(0.76; 1.23) | 0.53 | <0.001 |
| TN-4 | 35 | 0.91b(0.77; 1.08) | 0.23(-0.18; 0.64) | 0.76 | <0.001 |
| TN-5 | 39 | 1.35a(1.11; 1.64) | -0.90(-1.58; -0.23) | 0.66 | <0.001 |
| N | | | | | |
| All | 248 | 1.28(1.17; 1.40) | -0.37(-0.51; -0.22) | 0.49 | <0.001 |
| TN level | | | | | |
| TN-1 | 25 | 1.41a(1.05; 1.89) | -0.58(-1.12; -0.05) | 0.52 | <0.001 |
| TN-2 | 63 | 1.61a(1.35; 1.91) | -0.83(-1.19; -0.46) | 0.54 | <0.001 |
| TN-3 | 85 | 1.35a(1.17; 1.57) | -0.45(-0.69; -0.20) | 0.53 | <0.001 |
| TN-4 | 35 | 1.10b(0.85; 1.44) | -0.09(-0.45; 0.27) | 0.43 | <0.001 |
| TN-5 | 40 | 1.02b(0.81; 1.30) | -0.04(-0.35; 0.28) | 0.47 | <0.001 |
| P | | | | | |
| All | 231 | 0.94(0.88; 0.99) | 0.01(-0.02; 0.04) | 0.78 | <0.001 |
| TN level | | | | | |
| TN-1 | 21 | 1.60a(1.22; 2.09) | -0.06(-0.15; 0.03) | 0.68 | <0.001 |
| TN-2 | 55 | 1.01b(0.87; 1.17) | -0.02(-0.09; 0.05) | 0.71 | <0.001 |
| TN-3 | 80 | 0.95b(0.85; 1.07) | -0.01(-0.07; 0.05) | 0.73 | <0.001 |
| TN-4 | 34 | 0.90b(0.77; 1.05) | 0.04(-0.03; 0.11) | 0.81 | <0.001 |
| TN-5 | 41 | 0.88b(0.78; 1.00) | 0.05(-0.01; 0.10) | 0.85 | <0.001 |

**Supplementary Table 3** Sediment TN, sediment TP, water TN, water TP, SD (mean ± se) and scaling exponents (α_C_ (S-L/R), α_N_ (S-L/R) and α_P_ (S-L/R)) associated with five WTN levels (WTN≤0.5, 0.5<WTN≤1, 1<WTN≤1.5, 1.5<WTN≤2 and WTN>2 mg L^-1^ for TN-1, TN-2, TN-3, TN-4 and TN-5, respectively). Values of environmental factors and scaling exponents that have different letters are significantly different (p< 0.05) based on ANOVA and likelihood tests, respectively. SD, Secchi depth.

| α_C_ (S-L) | | | | | | |  | | | | |
| --- | --- | --- | --- | --- | --- | --- | --- | --- | --- | --- | --- |
| **TN level** | **Sediment**  **TN (mg g^-1^)** | **Sediment**  **TP (mg g^-1^)** | **Water TN (mg L^-1^)** | **Water TP (mg L^-1^)** | **SD (m)** | **αRMA (95%CI)** |  | | | | |
| TN-1 | 0.79±0.11c | 0.48±0.03c | 0.43±0.01e | 0.14±0.01b | 1.26±0.05a | 0.98b(0.74; 1.31) |  | | | | |
| TN-2 | 1.38±0.16bc | 0.58±0.03bc | 0.71±0.02d | 0.14±0.01b | 0.94±0.04b | 1.20b(0.98; 1.46) |  | | | | |
| TN-3 | 2.50±0.24a | 0.71±0.03ab | 1.23±0.01c | 0.19±0.01a | 1.05±0.08ab | 0.98b(0.84; 1.14) |  | | | | |
| TN-4 | 1.79±0.19abc | 0.66±0.05abc | 1.80±0.02b | 0.19±0.01a | 0.47±0.05c | 1.21b(1.01; 1.45) |  | | | | |
| TN-5 | 1.82±0.14ab | 0.77±0.08a | 2.70±0.10a | 0.17±0.01ab | 0.46±0.04c | 1.61a(1.30; 1.99) |  | | | | |
| α_C_ (S-R) | | | | | | |  |  |  |  | α_C_ (S-R) |
| TN-1 | 0.82±0.14b | 0.47±0.04b | 0.43±0.01e | 0.14±0.01a | 1.26±0.07a | 0.88b(0.70; 1.11) |  | | | | |
| TN-2 | 1.46±0.18ab | 0.57±0.03ab | 0.72±0.02d | 0.14±0.01a | 0.96±0.05a | 0.55c(0.45; 0.67) |  | | | | |
| TN-3 | 2.46±0.28a | 0.67±0.02a | 1.23±0.02c | 0.19±0.01a | 1.07±0.09a | 0.61c(0.53; 0.71) |  | | | | |
| TN-4 | 1.59±0.20ab | 0.61±0.05ab | 1.81±0.02b | 0.18±0.02a | 0.59±0.06b | 0.91b(0.77; 1.08) |  | | | | |
| TN-5 | 1.90±0.18ab | 0.59±0.07ab | 2.55±0.10a | 0.15±0.01a | 0.48±0.05b | 1.35a(1.11; 1.64) |  | | | | |
| α_N_ (S-L) | | | | | | |  |  |  |  | α_N_ (S-L) |
| TN-1 | 0.79±0.11c | 0.48±0.03c | 0.43±0.01e | 0.14±0.01b | 1.26±0.05a | 1.76a(1.42; 2.18) |  | | | | |
| TN-2 | 1.38±0.16bc | 0.58±0.03bc | 0.71±0.02d | 0.14±0.01b | 0.94±0.04b | 1.41a(1.21; 1.64) |  | | | | |
| TN-3 | 2.49±0.24a | 0.70±0.03ab | 1.23±0.01c | 0.19±0.01a | 1.06±0.08ab | 1.52a(1.35; 1.71) |  | | | | |
| TN-4 | 1.79±0.19abc | 0.66±0.05abc | 1.80±0.02b | 0.19±0.01a | 0.47±0.05c | 1.25b(1.05; 1.49) |  | | | | |
| TN-5 | 1.86±0.15ab | 0.77±0.08a | 2.70±0.10a | 0.17±0.01ab | 0.46±0.04c | 1.25b(1.06; 1.48) |  | | | | |
| α_N_ (S-R) | | | | | | |  |  |  |  | α_N_ (S-R) |
| TN-1 | 0.82±0.14b | 0.47±0.04b | 0.43±0.01e | 0.14±0.01a | 1.26±0.07a | 1.41a(1.05; 1.89) |  | | | | |
| TN-2 | 1.46±0.18ab | 0.57±0.03ab | 0.72±0.02d | 0.14±0.01a | 0.96±0.05a | 1.61a(1.35; 1.91) |  | | | | |
| TN-3 | 2.46±0.28a | 0.67±0.02a | 1.23±0.02c | 0.19±0.01a | 1.07±0.09a | 1.35a(1.17; 1.57) |  | | | | |
| TN-4 | 1.59±0.20ab | 0.61±0.05ab | 1.81±0.02b | 0.18±0.02a | 0.58±0.06b | 1.10b(0.85; 1.44) |  | | | | |
| TN-5 | 2.00±0.18a | 0.67±0.09a | 2.59±0.11a | 0.15±0.01a | 0.48±0.05b | 1.02b(0.81; 1.30) |  | | | | |
| α_P_ (S-L) | | | | | | |  |  |  |  | α_P_ (S-L) |
| TN-1 | 0.79±0.11c | 0.48±0.03c | 0.43±0.01e | 0.14±0.01c | 1.27±0.05a | 1.42a(1.25; 1.61) |  | | | | |
| TN-2 | 1.37±0.17bc | 0.57±0.03bc | 0.71±0.02d | 0.14±0.01bc | 0.96±0.04b | 1.27a(1.15; 1.40) |  | | | | |
| TN-3 | 2.49±0.24a | 0.70±0.03ab | 1.23±0.01c | 0.19±0.01a | 1.06±0.08ab | 1.23a(1.09; 1.40) |  | | | | |
| TN-4 | 1.82±0.19ab | 0.66±0.05abc | 1.80±0.02b | 0.19±0.01ab | 0.48±0.05c | 1.17b(1.02; 1.33) |  | | | | |
| TN-5 | 1.83±0.15ab | 0.77±0.08a | 2.71±0.10a | 0.17±0.01abc | 0.47±0.04c | 1.12b(1.01; 1.24) |  | | | | |
| α_P_ (S-R) | | | | | | |  |  |  |  | α_P_ (S-R) |
| TN-1 | 0.65±0.09b | 0.44±0.03b | 0.42±0.01e | 0.14±0.01a | 1.28±0.05a | 1.60a(1.22; 2.09) |  | | | | |
| TN-2 | 1.42±0.19ab | 0.55±0.03ab | 0.72±0.02d | 0.14±0.01a | 0.97±0.05a | 1.01b(0.87; 1.17) |  | | | | |
| TN-3 | 2.51±0.29a | 0.66±0.02a | 1.23±0.02c | 0.18±0.01a | 1.08±0.10a | 0.95b(0.85; 1.07) |  | | | | |
| TN-4 | 1.56±0.20ab | 0.60±0.05ab | 1.81±0.02b | 0.19±0.02a | 0.58±0.06b | 0.90b(0.77; 1.05) |  | | | | |
| TN-5 | 1.96±0.18a | 0.67±0.09a | 2.60±0.11a | 0.15±0.01a | 0.49±0.05b | 0.88b(0.78; 1.00) |  | | | | |

**Supplementary Table 4** Summary of reduced major axis (RMA) regression results for nutrient allocation among organs (e.g., log_10_stem C (or N, or P) = α*log_10_ leaf (or root) C (or N, or P) + β) of canopy former species grouped by five WTN levels (WTN≤0.5, 0.5<WTN≤1, 1<WTN≤1.5, 1.5<WTN≤2 and WTN>2 mg L^-1^ for TN-1, TN-2, TN-3, TN-4 and TN-5, respectively). Different letters indicate that scaling exponents are significantly different (p< 0.05) based on likelihood tests.

|  | **n** | **αRMA (95%CI)** | **βRMA（95%CI）** | **r^2^** | **p** |
| --- | --- | --- | --- | --- | --- |
| Scaling exponents of stem (C, or N, or P) to leaf (C, or N, or P) | | | | | |
| C | | | | | |
| All | 184 | 1.06(0.93; 1.20) | -0.16(-0.52; 0.20) | 0.20 | <0.001 |
| Canopy former | | | | | |
| TN-1 | 24 | 0.79b(0.56; 1.11) | 0.56(-0.17; 1.29) | 0.36 | 0.002 |
| TN-2 | 33 | 1.16a(0.83; 1.61) | -0.41(-1.43; 0.60) | 0.17 | 0.017 |
| TN-3 | 62 | 0.68b(0.54; 0.87) | 0.81(0.37; 1.24) | 0.12 | 0.007 |
| TN-4 | 30 | 0.91a(0.67; 1.24) | 0.21(-0.54; 0.96) | 0.34 | <0.001 |
| TN-5 | 35 | 1.23a(0.92; 1.65) | -0.62(-1.58; 0.34) | 0.30 | <0.001 |
| N | | | | | |
| All | 183 | 1.62(1.49; 1.75) | -1.17(-1.36; -0.99) | 0.71 | <0.001 |
| Canopy former | | | | | |
| TN-1 | 24 | 1.96a(1.65; 2.33) | -1.63(-2.11; -1.14) | 0.85 | <0.001 |
| TN-2 | 33 | 1.51b(1.30; 1.76) | -1.03(-1.36; -0.69) | 0.83 | <0.001 |
| TN-3 | 62 | 1.73a(1.51; 1.98) | -1.39(-1.73; -1.04) | 0.72 | <0.001 |
| TN-4 | 30 | 1.42b(1.17; 1.72) | -0.89(-1.31; -0.47) | 0.74 | <0.001 |
| TN-5 | 34 | 1.36b(1.11; 1.66) | -0.72(-1.14; -0.31) | 0.68 | <0.001 |
| P | | | | | |
| All | 180 | 1.05(0.99; 1.11) | -0.18(-0.21; -0.15) | 0.84 | <0.001 |
| Canopy former | | | | | |
| TN-1 | 24 | 1.35a(1.11; 1.64) | -0.25(-0.32; -0.17) | 0.81 | <0.001 |
| TN-2 | 32 | 1.05b(0.94; 1.17) | -0.18(-0.23; -0.12) | 0.91 | <0.001 |
| TN-3 | 61 | 1.14a(0.98; 1.33) | -0.24(-0.35; -0.13) | 0.64 | <0.001 |
| TN-4 | 29 | 0.99b(0.87; 1.13) | -0.20(-0.27; -0.12) | 0.89 | <0.001 |
| TN-5 | 34 | 1.02b(0.91; 1.14) | -0.13(-0.20; -0.06) | 0.90 | <0.001 |
| Scaling exponents of stem (C, or N, or P) to root (C, or N, or P) | | | | | |
| C | | | | | |
| All | 113 | 0.98(0.82; 1.15) | 0.07(-0.36; 0.50) | 0.19 | <0.001 |
| Canopy former | | | | | |
| TN-1 | 18 | 0.46(0.28; 0.74) | 1.43(0.84; 2.03) | 0.13 | 0.142 |
| TN-2 | 25 | 0.98a(0.69; 1.41) | 0.06(-0.88; 1.00) | 0.28 | 0.006 |
| TN-3 | 33 | 0.69(0.49; 0.98) | 0.81(0.17; 1.45) | 0.06 | 0.176 |
| TN-4 | 14 | 0.89(0.53; 1.49) | 0.30(-0.97; 1.57) | 0.24 | 0.072 |
| TN-5 | 23 | 0.83a(0.56; 1.24) | 0.44(-0.44; 1.33) | 0.20 | 0.032 |
| N | | | | | |
| All | 112 | 1.23(1.06; 1.43) | -0.31(-0.52; -0.09) | 0.38 | <0.001 |
| Canopy former | | | | | |
| TN-1 | 18 | 1.29a(0.95; 1.75) | -0.47(-0.97; 0.03) | 0.66 | <0.001 |
| TN-2 | 25 | 1.61a(1.17; 2.23) | -0.85(-1.50; -0.19) | 0.42 | <0.001 |
| TN-3 | 33 | 1.74a(1.31; 2.32) | -0.83(-1.39; -0.26) | 0.37 | <0.001 |
| TN-4 | 14 | 1.44(0.86; 2.42) | -0.42(-1.29; 0.45) | 0.26 | 0.06 |
| TN-5 | 22 | 0.76b(0.57; 1.01) | 0.28(0.01; 0.56) | 0.62 | <0.001 |
| P | | | | | |
| All | 106 | 0.85(0.76; 0.94) | 0.01(-0.02; 0.04) | 0.73 | <0.001 |
| Canopy former | | | | | |
| TN-1 | 15 | 1.72a(1.17; 2.53) | -0.05(-0.13; 0.03) | 0.57 | 0.001 |
| TN-2 | 23 | 0.97b(0.73; 1.29) | -0.03(-0.13; 0.08) | 0.59 | <0.001 |
| TN-3 | 32 | 0.86b(0.71; 1.04) | -0.02(-0.10; 0.06) | 0.73 | <0.001 |
| TN-4 | 14 | 0.82b(0.61; 1.10) | 0.03(-0.06; 0.12) | 0.77 | <0.001 |
| TN-5 | 22 | 0.80b(0.64; 0.99) | 0.06(0.02; 0.10) | 0.78 | <0.001 |

**Supplementary Table 5** Person correlation coefficients of water TN and other environmental factors. Pearson coefficients and p values are shown in lower left and upper right sections, respectively. SD, Secchi depth.

|  | Sediment TN | Sediment TP | Water TN | Water TP | SD |
| --- | --- | --- | --- | --- | --- |
| Sediment TN |  | <0.001 | 0.255 | <0.001 | <0.001 |
| Sediment TP | 0.215 |  | <0.001 | 0.001 | <0.001 |
| Water TN | 0.064 | 0.307 |  | 0.001 | <0.001 |
| Water TP | -0.228 | 0.191 | 0.181 |  | <0.001 |
| SD | 0.333 | -0.234 | -0.483 | -0.275 |  |

**Supplementary Table 6** Sediment TN, sediment TP, water TN and water TP (mean ± se) associated with three growth forms. Values of environmental factors that have different letters are significantly different (p< 0.05) based on ANOVA.

|  | all | Canopy former | Erect | Rosette |
| --- | --- | --- | --- | --- |
| Sediment TN (mg g^-1^) | 1.87±0.11 | 1.83±0.14a | 2.14±0.19a | 1.73±0.26a |
| Sediment TP (mg g^-1^) | 0.66±0.02 | 0.66±0.03ab | 0.74±0.05a | 0.60±0.03b |
| Water TN (mg L^-1^) | 1.37±0.04 | 1.40±0.06a | 1.36±0.12a | 1.32±0.06a |
| Water TP (mg L^-1^) | 0.17±0.01 | 0.18±0.01a | 0.12±0.01b | 0.20±0.01a |
